# The Role of GnIH in Biological Rhythms and Social Behaviors

**DOI:** 10.3389/fendo.2021.728862

**Published:** 2021-09-10

**Authors:** Chuin Hau Teo, Brandon Phon, Ishwar Parhar

**Affiliations:** Brain Research Institute, Jeffrey Cheah School of Medicine and Health Sciences, Monash University Malaysia, Bandar Sunway, Selangor, Malaysia

**Keywords:** GnIH, social behavior, circadian rhythms, reproductive rhythms, RFRP, reproductive activities

## Abstract

Gonadotropin-inhibitory hormone (GnIH) was first discovered in the Japanese quail, and peptides with a C-terminal LPXRFamide sequence, the signature protein structure defining GnIH orthologs, are well conserved across vertebrate species, including fish, reptiles, amphibians, avians, and mammals. In the mammalian brain, three RFamide-related proteins (RFRP-1, RFRP-2, RFRP-3 = GnIH) have been identified as orthologs to the avian GnIH. GnIH is found primarily in the hypothalamus of all vertebrate species, while its receptors are distributed throughout the brain including the hypothalamus and the pituitary. The primary role of GnIH as an inhibitor of gonadotropin-releasing hormone (GnRH) and pituitary gonadotropin release is well conserved in mammalian and non-mammalian species. Circadian rhythmicity of GnIH, regulated by light and seasons, can influence reproductive activity, mating behavior, aggressive behavior, and feeding behavior. There is a potential link between circadian rhythms of GnIH, anxiety-like behavior, sleep, stress, and infertility. Therefore, in this review, we highlight the functions of GnIH in biological rhythms, social behaviors, and reproductive and non-reproductive activities across a variety of mammalian and non-mammalian vertebrate species.

## 1 Introduction

Pituitary gonadotropins, stimulated by gonadotropin-releasing hormone (GnRH), did not have any known inhibitory hormone until the discovery of a novel RFamide neuropeptide [RFamide-related protein (RFRP)] in birds (1). Encoded by the *npvf* (neuropeptide VF) gene, RFRP dodecapeptide is also known as gonadotropin-inhibitory hormone (GnIH) because of its inhibitory effect on GnRH and gonadotropin release, shown for the first time in the Japanese quail *Coturnix japonica* (1). In the two decades since its discovery, GnIH has been identified in several mammalian (2–5) and non-mammalian species (4, 6). In general, GnIH and its orthologs perform similar functions across species, which is to regulate reproduction *via* the inhibition of GnRH-mediated gonadotropin release.

Three different RFamide-related proteins, RFRP-1, RFRP-2, and RFRP-3, orthologous to avian GnIH, have been identified from the mammalian brain; these proteins are cleaved from the propeptide NPVF (NPVF precursor) coded by the *npvf* gene (7). Among these mammalian GnIH orthologs, RFRP-1 and RFRP-3 contain the LPXRFa sequence, which is lacking in RFRP-2 (8). Subsequent studies have shown that the sequence previously considered to be the C-terminus of RFRP-2 is actually a part of the N-terminus of RFRP-3 (3, 9, 10), which means that RFRP-1 and RFRP-3 are the only orthologous GnIHs present in mammalian species (ovine, bovine, rodents, and primates). RFRP-3 has been shown to inhibit the synthesis and release of mammalian gonadotropin, demonstrating similar function and structural similarity to GnIH (5, 11, 12). In this review, GnIH and RFRP-3 will be used interchangeably, with RFRP-3 being used in particular when discussing the mammalian variant of the peptide.

From an evolutionary standpoint, peptides with a similar or homologous structure to GnIH have been isolated and identified in teleosts, birds, amphibians, reptiles, and mammalian species (12, 13). In each of these peptides, a similar C-terminal LPXRFamide (X = L or Q) sequence is observed, indicating evolutionary conservation of the amino acid motif within mammalian and non-mammalian vertebrates (14). While this suggests that LPXRFamides share a common trait in regulating pituitary functions and inhibiting GnRH, they have also diversified in their hypophysiotropic activities, particularly in non-mammalian vertebrates (15).

Internal factors such as sex steroids and external factors such as stress can regulate GnIH, which in turn may positively or negatively impact reproduction. GnIH-regulated gonadotropins [luteinizing hormone (LH) and follicle-stimulating hormone (FSH)] can also have an impact on GnIH itself—LH can decrease RFRP neuronal activity during the LH surge (16).

GnRH neurons are directly regulated by estrogen *via* estrogen receptor-β (ER- β) in mice (16–21), female hamster RFRP neurons express estrogen receptor-α (ER-α) (22), and estradiol-17β treatment decreases c-Fos activity in enhanced green fluorescent protein (EGFP)–GnIH neurons (23) in rats. The presence of estrogen receptors on GnIH neurons indicates that the GnIH system may also mediate reproductive activity *via* participation in the negative feedback loop of estrogen–GnRH.

More recently, *in vitro* hypothalamic GnIH neurons have been demonstrated to express glucocorticoid receptors (24), and Son et al. (25) have identified glucocorticoid responsive elements in the promoter region of the rat *npvf* gene that are receptive to corticosterone as well as corticosterone-stimulated recruitment of glucocorticoid receptors. These discoveries describe a molecular mechanism for the regulation of the GnIH system under stress that involves direct action by glucocorticoids. Another factor that controls GnIH is circadian rhythmicity. The cyclic nature of reproductive rhythms (26–31) suggests GnIH, being a reproductive molecule, changes in a seasonal and cyclic manner.

In this review, we highlight the functions of GnIH in reproductive rhythms, behaviors, and non-reproductive activities across a variety of mammalian and non-mammalian vertebrate species.

## 2 Distribution

To date, very few studies have been conducted on the localization, function, and binding of RFRP-1 and RFRP-2 independently of RFRP-3. In rodents, the distribution of RFRP-1 is highest in the hypothalamus, followed by the thalamus, midbrain, and optic nerve, with trace amounts in the hippocampus (3). GnIH peptides have been reported in the hypothalamus of various vertebrates across multiple species—bovine, rodent, avian, amphibians, and fish (32). Distribution studies for GnIH are extensive and have been covered in many reviews (14, 33, 34); as such, this paper will briefly summarize the results of those findings, with a major focus on fish, avians, and mammals.

In fish, GnIH mRNA is primarily localized in the nucleus posterioris periventricularis (NPPv) in the hypothalamus of goldfish (4), sockeye salmon (35), Indian major carp (36), and the tilapia (37). GnIH-immunoreactive fibers have been shown in the olfactory bulb, pituitary, and spinal cord (4, 35, 36). In the avians, GnIH is primarily found in the paraventricular nucleus (PVN) of the hypothalamus and GnIH fibers are seen in the median eminence and the diencephalic and mesencephalic regions (1, 12, 38).

GnIH neurons in rodents are located in highest density particularly within the compact dorsal and ventral regions of the dorsomedial nucleus of the hypothalamus (DMH) (22, 23). Numerous GnIH-immunoreactive fibers project into the hypothalamic and limbic structures, the diencephalic and mesencephalic regions, and come into close apposition with GnRH neurons (22, 39). In ovine species like the sheep, GnIH neuronal population is disperse throughout the DMH, PVN, and the medio-basal hypothalamus (2). In particular, GnIH cell bodies are observed in high density in the DMH, with their terminals projecting to the internal layer of the median eminence and to several midbrain regions including the diagonal band of Broca, preoptic area (POA), and the anterior pituitary (40). In general, GnIH is found in different parts of the brain depending on the species, though its presence in the hypothalamus and pituitary is common across mammals, avians, and fish (**Table 1**). The prominence of GnIH in the hypothalamus contributes to majority of its functions such as reproduction, feeding, anxiety, and social behaviour (**Figure 2**).

**Table 1:** Distribution of GnIH, GnIH-ir fibers, and GPR147 in various central and peripheral tissues.

Tissue	GnIH	GnIH-ir fibers	GPR147	Species	References
**Central tissues**					
Amygdala	-	+	+	Mammals	(14, 29, 30)
Hippocampus	-	+	+	Mammals	(14, 29, 30)
Nucleus posterioris periventricularis	+	+	+	Fish	(30)
Dorsomedial hypothalamus	+	+	+	Mammals	(14, 29, 30)
Paraventricular nucleus	+	+	+	Avian, mammals	(14, 29, 30)
Median eminence	-	+	+	Avian, mammals	(14, 29, 30)
Pituitary	+	+	+	Fish, avian, mammals	(14, 29, 30)
Olfactory bulb	–	+	+	Fish	(30)
Spinal cord	+	+	+	Fish, avian, mammals	(14, 29, 41)
**Peripheral tissues**					
Heart	+	–	+	Mammals	(42)
Gonads	+	–	+	Fish, Avian, Mammals	(43, 44)

+, regions where GnIH, GnIH-ir, or GPR147 has been found to be localized; -, regions where GnIH, GnIH-ir, or GPR147 has not been detected; GnIH, gonadotropin-inhibitory hormone.

## 3 Receptor Binding and Mechanism of Action

### 3.1 Specific Binding of GnIH to GPR147

G protein-coupled receptors (GPCRs) for GnIH were first identified by Hinuma et al. (7) where they found a cDNA that encoded a GPCR that responded to RFRP-1 and RFRP-3. The seven transmembrane receptor was named OTGT022 that corresponds to GPR147 (7). Bonini et al. (45), while investigating receptors for neuropeptide FF-amide (NPFF), a neuropeptide with a C-terminal PQRFa motif, discovered two GPCRs that interacted with NPFF, namely, NPFF1 (essentially GPR147) and NPFF2 (essentially GPR74) (45, 46). NPFFs bind to GPR74 with higher affinity in both COS-7 and HEK293 cell lines (45), indicating a possible difference in binding affinity between RFRP and NPFF with GPR147 and GPR74, respectively. RFRPs have about 100 times higher binding affinity for GPR147 than NPFFs, while NPFFs have about 10 times higher binding affinity to GPR74 than RFRPs (46–49).

Yin et al. (41) used a combination of 3′/5′ RACE with PCR primers based on the structure of the GPR147 from rats and cloned a cDNA encoding a GnIH receptor. They verified using a crude membrane fraction of COS-7 cell line transfected with the putative GnIH receptor cDNA that GnIH and GnIH-related peptides (GnIH-RPs) bind to GPR147 with high affinity, while non-amidated GnIH (GnIH-OH) fails to bind to GPR147 (41). Yin et al. (41) also used mammalian RFRP, chicken GnIH, GnIH-OH, and other neuropeptides lacking the C-terminal LPXRFa motif in competitive binding experiments to reveal that binding of GnIH to GPR147 relies on the critical LPXRFa C-terminal motif. In the competitive binding experiments, all GnIH orthologs successfully inhibited binding of avian GnIH, while GnIH-OH and the other neuropeptides without the C-terminal LPXRFa motif did not inhibit binding (41). The Scatchard plot analysis also showed that GPR147 had a single class of high-affinity binding sites (K_d_ = 0.752 nM) for GnIH and GnIH-RPs (41). Thus, it is well documented that GnIH mainly couples with GPR147.

Localization studies have shown GPR147 in brain areas such as the hypothalamus (50), pre-optic area (51), and spinal cord (52). GPR147 is also present in GnRH neurons of fish (6), avians (43), reptiles (44), amphibians (53), mammals (50), and humans (5). Furthermore, GPR147 is present in the pituitary (43) and in gonadotrophs of various non-mammalian and mammalian vertebrates. Furthermore, the expression of GPR147 has been shown in the testes (54) and ovaries (55) of many vertebrate species. These studies suggest that GnIH has a significant role in reproduction.

### 3.2 GnIH Mechanism of Action

GnIH receptors (GPR147) inhibit adenylate cyclase (AC) activity by coupling to G_αi_ protein (7), which has been shown in COS-7 cells transfected with GPR147. A decrease in G_αi_ mRNA levels follows RFRP exposure, suggesting that GPR147 might be coupled to G_αi_ (56). In another study, ovine RFRP treatment inhibited the increase in calcium levels generated by GnRH, which is essential for LH secretion (2). On the other hand, chicken GnIH treatment of GH3 cells transfected with GPR147 did not increase inositol phosphate and cAMP production, which are the main indicators for G_αs_ or G_αq_ coupling. This indicates that GPR147 does not couple to either G_αs_ or G_αq_. Co-stimulation of GH3 cells with GnIH and forskolin (FSK) significantly reduced cAMP CRE-luciferase activity in GH3 cells, revealing that GPR147 mainly couples with G_αi_ to inhibit GnRH activity (57).

**Figure 1:**
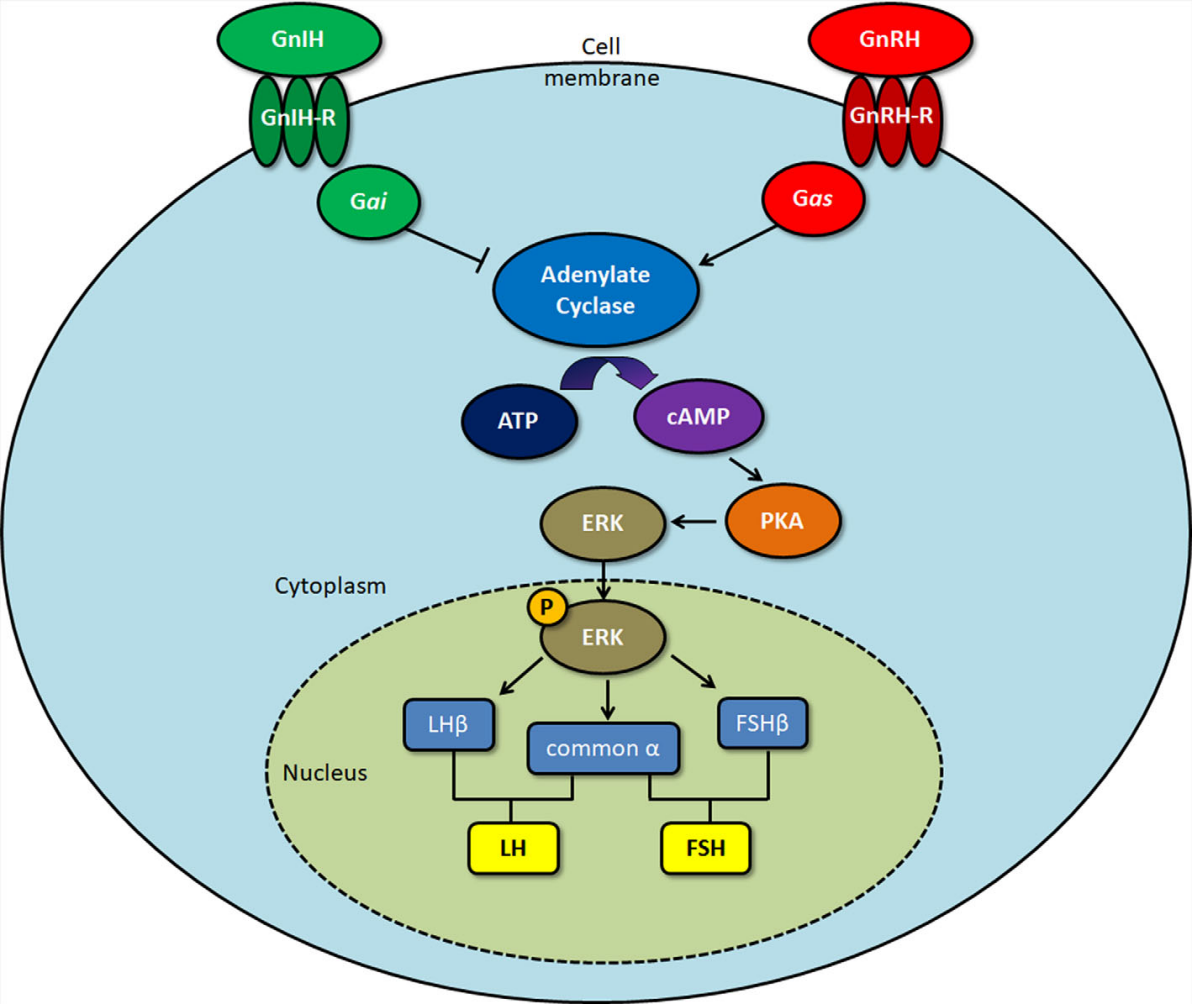
Signaling pathway of gonadotropin-inhibitory hormone (GnIH)/RFamide-related protein (RFRP)-3 upon binding to the GPR147 GnIH receptor. Protein kinase A (PKA), extracellular signal-regulated kinase (ERK). Gonadotropin-releasing hormone (GnRH) binds to the GnRH receptor, activating G*_as_* protein, which induces cAMP production. Upon binding to the GnIH receptor, G*_ai_* protein acts to inhibit GnRH-induced cAMP production, leading to a decrease in ERK activation. As phosphorylated ERK is involved in the transcription of the gonadotropin subunits LHβ, FSHβ, and common α, this ultimately results in downregulation of the gonadotropins that are formed, luteinizing hormone (LH) and follicle-stimulating hormone (FSH).

Son et al. (58) determined the GnIH/RFRP intracellular cell signaling pathway using a mouse gonadotrope (LβT2) cell line that exhibits all the characteristics of fully differentiated gonadotropes. FSK and GnRH-induced CRE-luciferase activity is significantly reduced by the adenylate cyclase inhibitor MDL (58). As mouse RFRP inhibits GnRH-induced increase in CRE-luciferase activity in a similar manner, this suggests that GnIH/RFRP directly inhibits GnRH-induced cAMP production (58). RFRP can also inhibit GnRH-stimulated extracellular signal-regulated kinase (ERK) phosphorylation elicited by GnRH in a mouse LBT2 cell line (58) and a mouse GnRH neuronal cell line (59). These experiments show that RFRP/GnIH specifically inhibits GnRH *via* the AC/cAMP/PKA pathway by coupling to G_αi_, preventing the activation of ERK1/2 signaling, that is important in the transcription of gonadotropins such as LHβ (**Figure 1**). While GnIH has also been shown to inhibit GnRH-induced increase in intracellular calcium (2), a process associated with the exocytotic release of the gonadotropins (60, 61) from the pituitary gland, the mechanism behind this action is yet undetermined.

## 5 Physiological Roles

**Figure 2:**
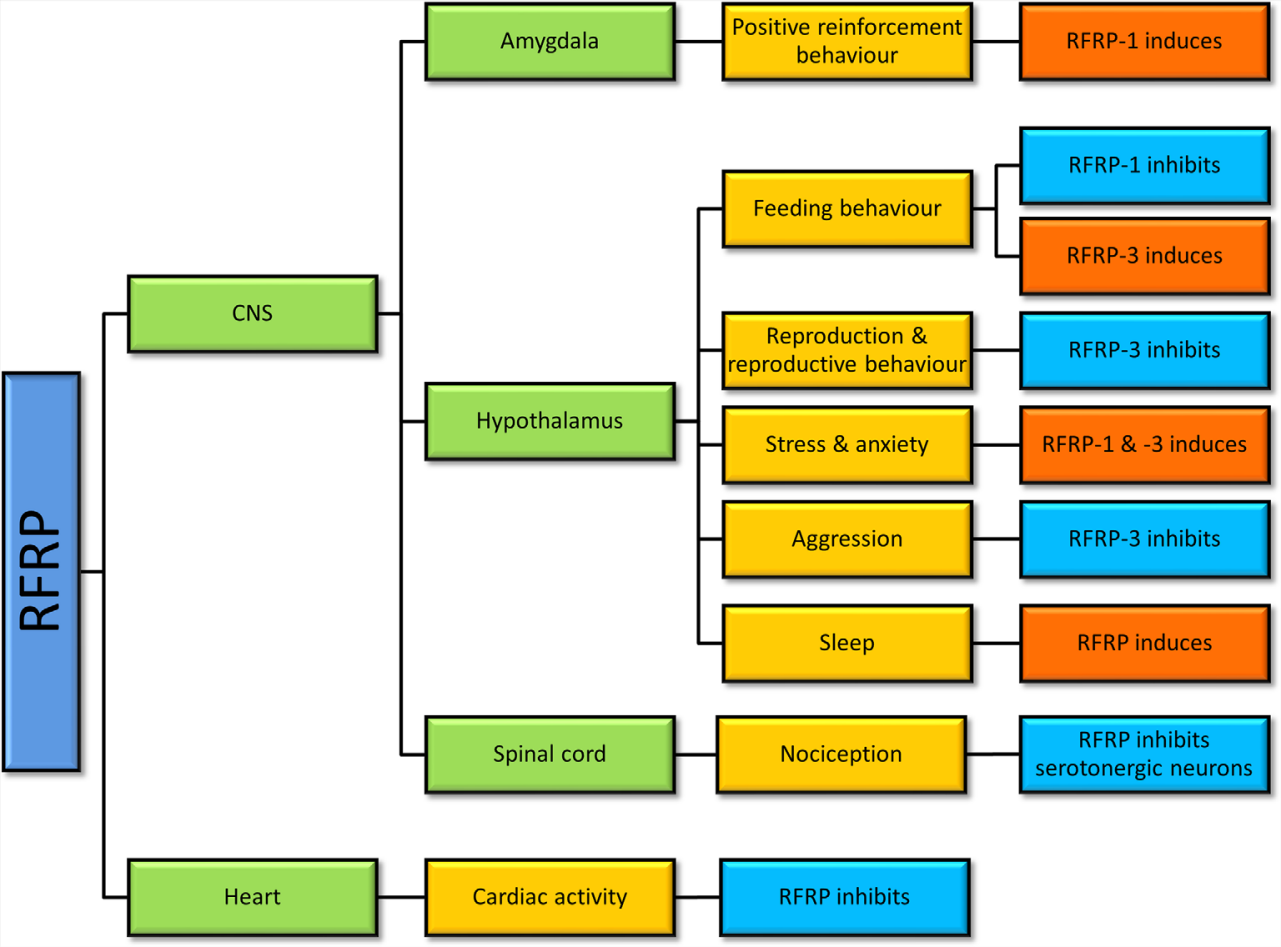
Physiological actions of gonadotropin-inhibitory hormone (GnIH)/RFamide-related protein (RFRP) in vertebrates. Studies of RFRP in vertebrates have determined that RFRP is involved in various physiological actions centering mainly around the hypothalamus, but not limited to that region.

A large body of data in non-mammalian and mammalian studies suggests that GnIH is involved in reproduction, reproductive rhythms, reproductive behaviors, social behaviors, circadian rhythms, and other physiological roles like nociception (47, 62, 63), learning (64), and cardiac activity (65) (**Figure 2**).

### 5.1 Reproduction

The involvement of GnIH in reproduction has been well conserved across vertebrate species even when mammalian species are administered with avian GnIH (66). There are various conditions such as sex, the process of gonadectomy, pubertal status, and duration of photoperiods that can lead to different effects of GnIH on LH/FSH secretion. Conflicting results have been shown across different experimental designs and animal models, which are summarized in **Table 2**.

**Table 2:** Summary of *in vivo* effects of GnIH/RFRP-3 injection on LH and FSH secretion.

Species	Condition	Injection	Effect	Reference
Tilapia	Female, adult	IP	Increases LH and FSH release	(37)
Sea bass	Female, adult	ICV	Decrease in plasma LH level	(67)
Goldfish	Female and male, adult	IP	Increase in LHβ and FSHβ mRNA during early to late gonadal recrudescence, reduced serum LH at early to mid-recrudescence	(68)
Sparrow	Female, adult	ICV	Decrease in plasma LH level	(69)
Quail	Male, adult	IV	Decrease in LHβ, FSHβ mRNA expression and serum LH level	(70)
Rat	Female, adult, OVX	IV	Gradual decrease in plasma LH level	(71)
Rat	Female, adult, OVX	Acute ICV	No significant suppressive effect on the mean concentration and pulsatile secretion of LH	(71)
Rat	Female, adult, OVX, low dose of estradiol	Acute ICV	No significant suppressive effect on the mean concentration and pulsatile secretion of LH	(72)
Rat	Female, adult, OVX, high dose of estradiol	Chronic ICV	Slight but insignificant decrease in LH concentration	(72)
Rat	Female, adult, GNX	Acute ICV	Decrease in circulating LH level but no changes to the circulating FSH level	(73)
Rat	Male, adult, GNX and Intact	Acute ICV	Decrease in circulating LH and FSH level	(73)
Rat	Male, adult, GNX	IV	Moderate decrease in circulating LH and FSH level	(73)
Mouse	Prepubescent, female, intact; prepubescent, female, OVX, E2 replacement; Adult, female, OVX; Adult, female, OVX, E2 replacement	Acute ICV	Decrease in LH concentration with no changes to FSH concentration	(74)
Mouse	Prepubescent, female, OVX	Acute ICV	No changes in LH concentration	(74)
Mouse	Male, adult, GNX and intact	Acute ICV	Stimulates secretion of LH	(75)
Mouse	Female, adult, E2-negative feedback conditions	Acute ICV	No effect on LH secretion	(75)
Mouse	Female, adult, preovulatory-like surge	Acute ICV	Decrease in LH secretion	(75)
Mouse	Adult, intact, male or female, diestrus or proestrus	IP	No changes in LH concentration	(75)
Bovine	Male, 5 months old, castrated	IV	Decrease in LH pulse frequency with no changes to the concentration	(76)
Syrian hamsters	Male, adult, LP and SP	Acute ICV	Increase in plasma LH and FSH levels	(77)
syrian hamsters	Female, adult, OVX, LP	Acute ICV	No changes in LH concentration	(77)
syrian hamsters	Male, adult	IP	Insignificant inhibition of basal LH levels	(78)
syrian hamsters	Female, adult, LP	Acute ICV	Decrease in basal LH concentration on the day of proestrus	(79)
syrian hamsters	Female, adult, SP	Acute ICV	No effect on the basal LH concentration	(79)
syrian hamsters	Female, adult LP	Chronic ICV	Decrease in LH concentration	(79)
syrian hamsters	Female, adult, SP	Chronic ICV	Increase in LH concentration	(79)
LVG hamsters	Female, adult, OVX	Acute ICV and IP	Decrease in LH concentration	(22)
Ovine	Female, adult OVX	IV	Decrease in LH pulse amplitude but no effect on FSH secretion	(2)
Ovine	Intact; OVX, estrogen induced LH surge	IV	Decrease in pulse amplitude as well as concentration	(80)
Ovine	Female, adult, OVX, estrogen-induced LH surge; Female, adult, Intact, acyclic	IV	No changes in LH secretion or plasma LH concentration	(81)
Mare	Intact, mature, breeding season	IV	No changes to the LH pulse amplitude, frequency, and concentration	(82)
Human	Female, adult, postmenopause	IV	Significant decrease in LH secretion	(83)
Human	Male, adult	IV	No changes in LH secretion	(83)

GnIH, gonadotropin-inhibitory hormone; RFRP, RFamide-related protein; LH, luteinizing hormone; FSH, follicle-stimulating hormone; OVX, ovariectomized; GNX, gonadectomized; IV, intravenous injection; ICV, intracerebroventricular injection; IP, intraperitoneal injection; LP, long photoperiod; SP, short photoperiod.

The expression of RFRP-1 is different between adult female and male rats. While RFRP-1 neurons and immunoreactive fibers remain unchanged in male rats during puberty, an increase is seen in post-pubertal female rats, suggesting a role for RFRP-1 in the regulation of the estrous cycle (84). RFRP-1 injections to mice induce estradiol release in a dose-dependent manner, which stimulates increased steroidogenesis in the ovaries (85). However, proliferating cell nuclear antigen (PCNA), caspase-3, and cleaved poly (ADP-ribose) polymerase (PARP) expression are significantly reduced, suggesting that RFRP-1 directly acts to inhibit folliculogenesis in the ovary (85).

### 5.2 Biological Rhythms

#### 5.2.1 Reproductive Rhythms

***Fish:*** A clear example of reproductive rhythms in fish can be seen in the grass puffer fish, *Takifugu niphobles*. In particular, GnIH levels within the diencephalon vary, and the expression peak shifts depending on whether the fish were placed in a natural light/dark condition or in a constant dark condition (86). Melatonin has circadian expression in the diencephalon; when administered intraperitoneally to the grass puffer fish, melatonin increases the expression of GnIH, which shows the regulation of GnIH by melatonin and the circadian clock (86). A recent study observed the effect of various spectra of LED lights on reproductive hormones in goldfish brain cells including GnIH neurons (87). In this *in vitro* study, goldfish brain cells were exposed to red, green, and blue LED light with white fluorescent light used as control; it was found that GnIH expression was significantly lower in the cells exposed to green and blue LED light and in groups treated with melatonin (87). Furthermore, while melatonin receptors and melatonin levels were elevated at night and decreased during the day, they were expressed at relatively higher levels in groups exposed to white fluorescent and red LED light compared to groups exposed to green and blue LED light (87). Choi et al. (87) hypothesized that circadian expression of melatonin interacted with RFRP and kisspeptin, which in turn control reproductive hormone levels that induce sexual maturation in fish.

In the European sea bass (*Dicentrarchus labrax*), pinealectomy on males resulted in lowered expression of GnIH in the mid-hindbrain (88). GnIH and GnIH receptor expression was also significantly reduced during reproductive seasons when compared to resting seasons (88). A long-term study on the effect of temperature on sea bass development over a period of a year demonstrated the presence of circadian rhythms in the daily expression of GnIH and GnIH receptors; at early developmental stages, GnIH and GnIH receptors were more highly expressed in the day, while more mature sea bass expressed a shift to higher nocturnal levels (88).

In the cinnamon clownfish, intraperitoneal injections of GnIH increased melatonin levels in the fish, confirming that GnIH, besides its role in suppressing GnRH and sexual maturity of the clownfish, also affects melatonin production (89). This suggests that melatonin and GnIH may interact by reciprocally stimulating each other.

***Avian:*** Photoperiod-dependent expression of GnRH and GnIH has been shown to regulate seasonal reproduction in the Eurasian tree sparrow. GnIH mRNA and GnIH-immunoreactive neurons increased significantly during the non-breeding season, and exposure to short days (SDs) induced higher GnIH expression compared to long day (LD) exposure, a change that happened regardless of the sampling month (90). In another study, sparrows were entrained to resonate with light–dark cycles, where a constant 6-h light phase was combined with a dark phase that served to vary the period of the light–dark cycles by 12-h increments (91). It was found that specific increments were interpreted by the birds’ circadian system as SD or LD. Resonance cycles that were read as LD would see testicular growth and reduction of GnIH, while resonance cycles read as SD would see significant increase in GnIH expression (91). This suggests the presence of an endogenous circadian rhythm regulating photoperiodic expression of GnIH. In other words, constant 6 h of light meant that the resonance cycle was read as SD or LD depending on whether the light was present on the photoinducible or non-photoinducible phase of the endogenous circadian cycle (91).

***Mammals:*** Mason et al. (92) found that Syrian male hamsters that were exposed to SD photoperiods exhibited decreased GnIH immunoreactivity and mRNA expression in comparison to those exposed to LD photoperiods. DMH containing GnIH neurons may serve as a mediator for melatonin action to control gonadotropic release (93). Conversely, since the suprachiasmatic nucleus (SCN) itself is a major target for melatonin action (94), its projections to GnIH neurons in the DMH may be another possible pathway of GnIH regulation through a photoperiod-related circadian system. Ubuka et al. (95) showed lower GnIH mRNA expression in Siberian hamsters exposed to SD photoperiods compared to hamsters exposed to LD photoperiods. While GnIH has inhibitory effect in mammalian species such as rodents and humans (66), it can play a different role in seasonal reproduction. Elevated GnIH expression in LD breeders such as hamsters (96) appears to have a stimulatory effect on the reproductive axis, increasing the secretion of LH (77, 79). Increased GnIH expression during LD photoperiods is conserved across multiple mammalian species, as SD breeders such as sheep (97) and goats also exhibit elevated GnIH during LD (98). As GnIH plays an inhibitory role in SD breeders (97), this shows that while photoperiod-dependant expression of GnIH is conserved, its regulatory effect downstream has evolved differently to induce reproductive axis stimulation in LD breeders and inhibition in SD breeders.

#### 5.2.2 Feeding Rhythms

***Avian*:** Intracerebroventricular (ICV) injection of GnIH into Peking duck resulted in a decrease in the plasma LH concentration and an increase in the food intake in Peking duck (80, 99). Feeding behaviors were also regulated by orexigenic peptides in the hypothalamus. Neuropeptide Y (NPY) is an orixegenic peptide produced by appetite-regulating cells and is known to stimulate food intake while pro-opiomelanocortin (POMC) is a precursor protein that gives rise to peptide derivatives that are associated with satiety (100–102). Red-headed buntings demonstrate a seasonal increase in cell optical density in NPY neurons in the DMH (103). As NPY fibers have been shown to be structurally associated with GnIH neurons in the Indian weaver bird (104), any change in NPY may in turn affect GnIH expression. An example may be found in a study where adult male Albert’s Towhees songbirds were food restricted during the photo-induced reproductive development phase (105). A 4-week food restriction significantly increased NPY cell number and, at the same time, decreased GnIH perikarya area (105). The decrease in GnIH perikarya area coincided with a decrease in plasma LH (105), suggesting that the heightened activity of the NPY system increased secretion of GnIH and, subsequently, inhibition of LH. In chickens (*Gallus gallus*), ICV injections of GnIH elevates food intake and increases neuronal activity in the lateral hypothalamic area, along with an increase in melanin-concentrating hormone and NPY expression and a decrease in POMC expression (102). In contrast to appetite stimulation by GnIH, ICV injection of RFRP-1 in chicks reduces food and water intake (106).

***Mammals*:** Studies on the effect of RFRP-3 infusion in mammals, particularly in mice and sheep, also saw an increase in food intake that was consistent with the results observed in birds (80). The study by Clarke et al. (80) observed the role of RFRP-3 in acting as a switch for preference between feeding and reproductive activity in sheep and rats. The clear opposition between feeding and reproductive function appears to suggest that high levels of RFRP-3 activity favors feeding over reproduction. It is possible that seasonal breeders such as sheep may exhibit reduced feeding behavior during mating seasons due to RFRP-3. On the other hand, injection of RFRP-1 into the central nucleus of the rat amygdala caused a decrease in food intake (107). As an NPFF receptor selective antagonist eliminated the effect in that same study, this demonstrated that the reduction in food intake was due to a receptor-linked effect in the amygdala (107).

Since feeding can be rhythmic in nature and is associated with GnIH regulation (80, 102) (**Figure 2**), the circadian nature of GnIH in the hypothalamus needs further investigation. Furthermore, as RFRP-1 inhibits appetite in contrast to RFRP-3’s stimulation of feeding behavior (80, 99, 106, 107), further differences in their other physiological activities may exist.

### 5.3 Reproductive Behavior

***Fish:*** Although GnIH has been shown to play a role in reproductive function in fish (88), its influence on fish mating behavior remains unclear. While a recent study has demonstrated that the Nile tilapia experiences upregulation of GnIH due to defeat in territorial fights (108), the role of GnIH in reproductive behavior such as courting and brooding is yet unknown.

***Avians*:** GnIH is directly responsible for the regulation of mating behavior in avians. In birds, GnIH neurons extend their projections to the periaqueductal central gray (PAG) and POA, signifying their possible role in the regulation of socio-sexual behaviors (109). Central administration of GnIH inhibits copulation in white-crowned sparrows (69). When infused directly into the brain, GnIH binds specifically to areas in the diencephalon and the midbrain where cGnRH-II-immunoreactive neurons reside (69). Since GPR147 is expressed in GnRH-II neurons, it can be speculated that GnIH suppresses sexual behaviors in birds by suppressing the activity of GnRH-II neurons (110). Silencing the GnIH-encoding *npvf* gene using RNA interference reduces the rest-time and increases spontaneous production of complex vocalizations and agonistic vocalizations in male and female white-crowned sparrows, which are part of mating behavior (111). Heightened vocalization (song production) in male birds in response to novel male songs is associated with an increase in locomotor activity, which suggests a greater degree of central nervous system (CNS) arousal when GnIH is inhibited (111). Furthermore, the activity of the male birds is positively correlated to the numbers of GnRH-I and GnRH-II neurons, which are in close proximity to GnIH-immunoreactive neuronal fiber terminals. This provides further evidence of inhibition of sexual arousal in white-crowned sparrows through the decrease in GnRH-I and GnRH-II neuronal activities (111). The intense RFRP-immunoreactive fiber density in the ventral tegmental area (VTA) of the female sparrows also suggests that the inhibitory role for GnIH in arousal of the CNS is not sex-limited (111).

***Mammals*:** ICV injections of RFRP-3 induced a decrease in plasma LH and a significant inhibition of sexual behavior (39). Female Syrian hamsters treated with RFRP-3 show decreased sexual motivation and vaginal scent marking but had no effect on copulatory behaviors. An increased expression of c-Fos was induced by RFRP-3 in the medial POA, bed nucleus of the stria terminalis, and the medial amygdala, all of which are part of the circuitry for female sexual behavior (112). Chronic immobilization stress-induced elevation of GnIH in rats decreases sexual behavior (42), pregnancy rate, and embryo resorption. These negative reproductive effects can be effectively reversed by silencing RFRP-3 using sh-RNA during stress (42). The effect can also be replicated in males, as a decrease in male sexual behavior in rats was reported upon central administration of RFRP (39).

## 6 Social Behavior

### 6.1 Aggression

***Avians*:** While there is no study directly linking GnIH to aggression in piscine species, GnIH is known to influence aggressive behavior in birds. Central administration of GnIH into male quails significantly inhibits their aggressive behavior, and GnIH RNA interference significantly increases aggression in quails (113).

***Mammals*:** In mice, RFRP-3 neurons project to neural loci regulating aggression in addition to neuroendocrine cells controlling the production of testosterone (114). Aggressive encounters between male mice reduce RFRP/c-Fos co-localization in anteroventral periventricular kisspeptin neurons (114). As RFRP acts as a negative regulator of the reproductive axis in mice by inhibiting GnRH, lowered RFRP-3 activity results in increased reproductive axis function, which facilitates an increase in testosterone and aggressive behavior (114). Furthermore, it has been shown that consumption of a large amount of soya bean leads to the suppression of GnIH and reduces aromatase activity, which is responsible for converting testosterone into neuroestrogen, leading to increased aggression in mice (115).

### 6.2 Stress and Anxiety

It is known that dysfunction of the hypothalamic–pituitary–adrenal (HPA) axis dysregulates the serotonergic system (116). GnIH is closely linked to the HPA axis since GnIH neurons express glucocorticoid receptors (117), and *in vitro* experiments show that glucocorticoids stimulate GnIH mRNA expression (24). Stress can lead to anxiety through GnIH’s action on the serotonergic system.

***Fish*:** In the cinnamon clownfish, cortisol treatment simulated an increase in GnIH mRNA but decreased GnRH as well as lowered circulating levels of LH and FSH (118), which suggests that glucocorticoids directly increase GnIH expression. In addition, a recent experiment on the male Nile tilapia showed that acute stress inflicted by social defeat increased GnIH mRNA levels in the NPPv and hypothalamus, as well as GPR147 mRNA in the pituitary. However, corticotropin-releasing hormone (CRH) and adrenocorticotropic hormone (ACTH) were not elevated, which suggests that GnIH may be directly affected by glucocorticoid signaling without an increase in CRH and ACTH levels (108).

***Avians*:** Capture–handling was used to examine the role of stress in manipulating the number of GnIH neurons in the hypothalamus of adult male and female house sparrows (119). More GnIH-positive neurons were seen in the fall as opposed to during the spring, where it is the start of the breeding season. A significant increase in GnIH-positive neurons was detected in stressed birds during the spring compared to those during the fall season (119). These observations suggest that the regulation of GnIH by stress changes over the annual reproduction cycle (119). Whether the regulation of GnIH during stress is through glucocorticoid receptors expressed in GnIH neurons or indirectly through other neuropeptides remains unknown.

***Mammals*:** Administration of RFRP-1 induces ACTH and oxytocin release in rats, facilitating an anxiogenic effect. The same effect is observed with RFRP-3, suggesting a similar function for both RFamide peptides (120). These anxiogenic effects of RFRP-1 and RFRP-3 are in stark contrast with the antidepressive effects of RFRP-1 reported in a mouse forced swim test (121). Selective serotonin reuptake inhibitor (SSRI) citalopram, an antidepressant, increases GnIH neuronal numbers in the DMH and fiber projections to the POA (122). As these brain regions are involved in reproduction, there are clear links between GnIH, the reproductive axis, and the serotonergic system (122).

**Figure 3:**
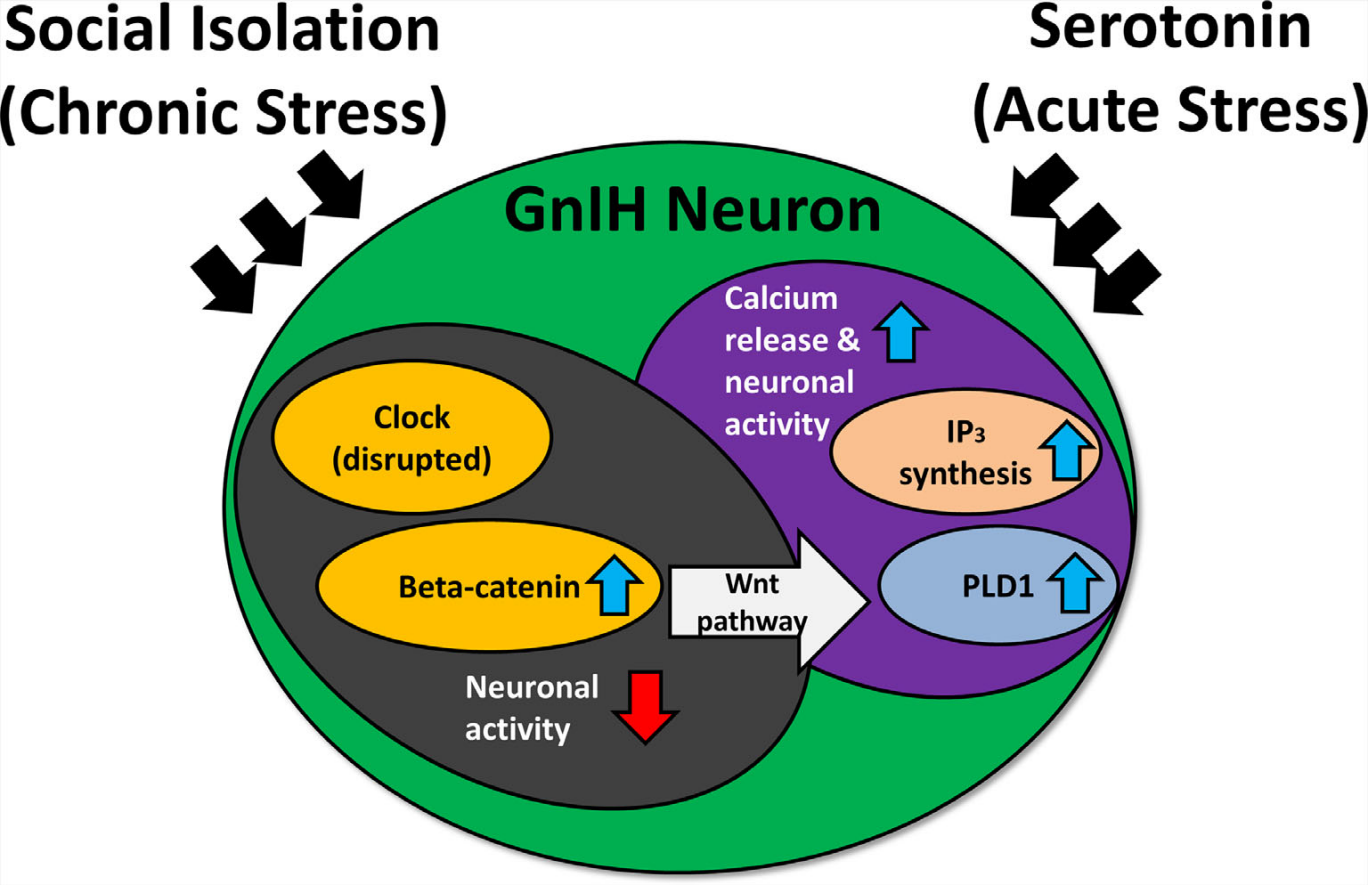
The effect of social isolation and serotonin on CLOCK expression and neuronal activity of gonadotropin-inhibitory hormone (GnIH) neurons. IP_3_, inositol trisphosphate; PLD1, phospholipase D1. Blue arrows indicate an increase, while red arrows indicate a decrease. Chronic stress may disrupt the expression of clock genes, inducing an increase in beta-catenin while lowering neuronal activity. The heightened levels of beta-catenin activate the Wnt pathway, which can upregulate PLD1 levels. This results in increased IP_3_ production, triggering a heightened calcium response under acute stress and subsequently elevating neuronal activity.

In adult rodents, immobilization stress or treatment with glucocorticoid receptor agonist, dexamethasone, increases RFRP-3 protein and inhibits hypothalamic-pituitary-gonadal (HPG) activity (117, 123). On the other hand, adrenalectomy blocks the increase in RFRP-3 expression brought about by stress. Stress exposure increases c-Fos expression in GnIH neurons of the DMH, and direct administration of RFRP-3 induces anxiety-like behavior in rats (120). More recently, social isolation in rats has been shown to disrupt the expression of circadian locomotor output cycles kaput (CLOCK) protein and beta-catenin, a protein known to control the circadian system and implicated in social isolation-induced depression (124). Furthermore, responsiveness of GnIH neurons to serotonin differs in relation to beta-catenin expression levels (125). Thus, chronic stress-induced RFRP-3 expression may disrupt circadian rhythmicity *via* beta-catenin and the serotonergic system (**Figure 3**). Under chronic stress, clock genes may experience disruption (124), inducing an increase in beta-catenin while lowering neuronal activity (125). Beta-catenin is a vital part of the Wnt signaling pathway—activation of this pathway elevates phospholipase-D1 (126), which is connected with elevated inositol trisphosphate and calcium release (127, 128). This could leave the cell more sensitive to acute stress. Hypothalamic RFRP-3 cells express glucocorticoid receptors (117, 119), and glucocorticoid response elements are present in the promoter region of the rat RFRP-3 gene (24). This could contribute, in part, to the mechanism of regulation of GnIH under stressful situations.

## 7 Sleep

***Fish*:** Circadian influence on GnIH has been suggested for sleep. The *npvf* gene encoding for RFRP-1 and RFRP-3 has been associated with sleep in larval zebrafish. Overexpression of RFRP *via* a heat shock-inducible promoter drastically increases sleep duration for the zebrafish (129). However, when RFRP is overexpressed in the middle of the day, the sleeping pattern of the night is unchanged, suggesting that there are other circadian components that prevent sleep from occurring early (129).

In larval zebrafish, increasing the expression of either RFRP-1 or RFRP-3 *via* a transgene reduced locomotor activity but did not increase sleep, while overexpression of RFRP-2 significantly reduced locomotor activity and increased sleep (129). However, the greatest impact on inducing sleep was observed with the overexpression of a combination of any two of the three RFRPs, demonstrating results similar to those of the wild type (129). Furthermore, stimulation of GnIH neurons produced activity levels similar to that normally observed at night and suppressed neuronal activity throughout the brain. Lastly, suppression of GnIH neurons also promoted wakefulness in the larvae (129).

The control of sleep by GnIH functions through the serotonergic raphe nuclei, since GnIH neurons are densely innervated by serotonergic projections from the raphe nuclei in zebrafish larvae (130). Optogenetic stimulation of RFRP neurons activated serotonergic neurons in the inferior raphe, and ablations of the serotonergic neurons of the raphe nuclei caused sleep time to remain unchanged even when GnIH neurons were stimulated (130). Larval zebrafish with intact raphe nuclei continued to exhibit the increased sleep time observed in the previous study (129), which suggests that GnIH acts upstream of serotonin to modulate sleep levels and wakefulness (130).

## 8 Conclusion

GnIH has been isolated and sequenced in a wide range of mammalian and non-mammalian vertebrate species. RFRP-1, RFRP-2, and RFRP-3 in the mammalian brain have been identified as orthologous to the avian GnIH. In the brain, the hypothalamus is the main region where GnIH neurons are located in all vertebrate species. However, GnIH neurons are also located outside the hypothalamus in some species. GnIH binds to its GPCR, GPR147, which has a widespread distribution in the brain including GnRH neurons. GnIH regulates reproduction by inhibiting GnRH and pituitary LH and FSH levels. In addition, in most vertebrate species, GnIH also regulates aggression, sleep, mating behavior, anxiety-like behavior, feeding behavior, non-reproductive social behavior, as well as stress-related infertility. Photoperiod-dependent fluctuation in GnIH has an important role in the circadian biology of reproduction. The majority of the published studies focus on RFRP-3 and its avian ortholog GnIH. On the other hand, functions of RFRP-1 have been less explored. As there are indications of possible functional dissimilarities between RFRP-1 and RFRP-3, elucidating the functions of RFRP-1 can be a promising avenue for future studies.

## Author Contributions

TC, BP, and IP wrote the manuscript. All authors contributed to the article and approved the submitted version.

## Conflict of Interest

The authors declare that the research was conducted in the absence of any commercial or financial relationships that could be construed as a potential conflict of interest.

## Publisher’s Note

All claims expressed in this article are solely those of the authors and do not necessarily represent those of their affiliated organizations, or those of the publisher, the editors and the reviewers. Any product that may be evaluated in this article, or claim that may be made by its manufacturer, is not guaranteed or endorsed by the publisher.
